# Reassessing magnetic tunnel junction detectability for ultrasensitive sensing using small-field sensitivity and Jiles–Atherton modeling

**DOI:** 10.1088/1361-6463/ae802f

**Published:** 2026-07-02

**Authors:** Benjamin J Brown, Liam K Mitchell, Hongzhou Yu, Gang Xiao

**Affiliations:** Department of Physics, Brown University, Providence, RI 02912, United States of America

**Keywords:** magnetic tunnel junction, Jiles–Atherton model, magnetic hysteresis, magnetic sensor, magnetometry, small-field sensitivity

## Abstract

Magnetic tunnel junction (MTJ) sensors are attractive for detecting extremely small magnetic fields, yet their performance is often quantified using sensitivities derived from major loops or larger-field minor loops which inherently include hysteretic contributions. As a result, these conventional metrics significantly overestimate detectability by including irreversible processes that vanish in the small-field limit. Here, we systematically measure the low-field response of uniaxial MTJs under both ac and dc magnetic excitation and demonstrate a distinct transition from hysteretic to fully reversible behavior as the field amplitude is reduced. By adapting the Jiles–Atherton model to analyze MTJ conductance, we establish a unified framework that captures magnetization processes down to nanotesla range, yielding quantitative parameters that resolve reversible and irreversible dynamics with excellent fidelity. In the reversible regime, the sensitivity converges to a constant value of 0.55%Oe$ ^{-1}$, defining an intrinsic, history-independent response of the MTJ. Importantly, this is less than half of the 1.2%Oe$ ^{-1}$ obtained from higher-field minor loop estimates but is physically representative of the sensing response in low-field operation. The constant and intrinsic sensitivity corresponds to the true value of detectability, confirmed by noise spectral density measurements under low-field ac excitation. These results establish a quantitative framework for MTJ evaluation that emphasizes intrinsic, hysteresis-free performance, providing both a realistic basis for calculating field detectability and a general methodology for probing magnetization in micron- and nano-scale ferromagnets.

## 1. Introduction

Magnetic tunnel junctions (MTJs) are fundamental building blocks in spintronic technology, essential not only for their roles in magnetic random-access memory and spin-transfer-torque devices, but also for their exceptional magnetic-field sensitivity [1–3]. This sensitivity has driven their increasing use in applications ranging from biomedical diagnostics [4–6] and nondestructive evaluation [7, 8] to geological exploration [9–11]. However, their operational performance is often obscured by magnetic hysteresis [12] within the ferromagnetic free layer, which complicates quantitative evaluation. Conventional sensitivity metrics derived from hysteretic loops inherently include irreversible domain processes, leading to inflated response estimates and overoptimistic predictions of detectability when used in noise-equivalent field calculations.

While previous efforts have reduced hysteresis through structural engineering, exchange-bias tuning, and material optimization [13–16], hysteresis collapses naturally in the small-field limit used for ultrasensitive applications. Despite this, detectability, the key figure-of-merit of a sensor, is frequently overestimated by using sensitivity values before this collapse occurs. This discrepancy is critical because the utility of detectability as a performance metric relies on the assumption that the sensitivity used in its calculation accurately represents the sensor’s response at the noise floor.

In this study, we systematically characterize the ac and dc magnetoresistive responses of uniaxial MTJs [17] over a wide range of excitation amplitudes, directly mapping the transition from hysteretic to fully reversible magnetization dynamics. At sufficiently small amplitudes approaching the noise floor, the magnetic sensitivity converges to a constant value, that defines the intrinsic dynamics of the free layer. This low-field sensitivity yields a detectability of 10 nT $\sqrt{\mathrm{Hz}}^{-1}$ at 1 Hz. While this value is higher than those derived from inflated hysteretic loop estimates, it is confirmed as the true physical limit of the device by our direct ac-excitation noise measurements.

To capture this transition, we employ the Jiles–Atherton (JA) model [18], a phenomenological framework that explicitly accounts for various hysteretic magnetic processes in magnetization-field loops. The model incorporates both irreversible domain-wall pinning and reversible magnetization rotation within a unified analytical formulation. While alternative frameworks like the Preisach model offer distinct advantages for complex loop fitting, the JA model provides a direct connection to the underlying physical mechanisms of the film. Furthermore, its native structure maps directly from differential susceptibility ($\mathrm{d}M/\mathrm{d}H$) to the sensor’s sensitivity, the primary experimental variable under investigation here. Owing to its balance between physical interpretability and computational efficiency compared to micromagnetic simulations, the JA model has been widely employed to characterize the hysteretic behavior of both soft and hard magnetic materials [19–21]. Although originally developed for bulk ferromagnets, it has been successfully used to describe thin-film and nanostructured systems [22–24], but its application has been limited in the low-field regime due to low signal in conventional magnetometry.

Here, we extend this framework by applying the JA model directly to MTJs in the low-field limit, establishing a quantitative link between tunneling conductance variation and the underlying magnetization processes in the free layer. By exploiting the high signal of MTJs in this regime, where the resolution of conventional magnetometers is fundamentally limited, we validate the JA model at the nanotesla scale by extracting a coherent set of model parameters that describe both dc minor loops and field-dependent ac sensitivities simultaneously. The extracted parameters describe the reversible sensitivity, hysteresis loss, and field-dependent susceptibility, providing a rigorous physical basis for interpreting MTJ behavior in the low-field regime. This approach suggests a route toward quantitative, spintronic-based magnetometry applicable to both sensor development and the study of magnetic materials under ultrasensitive conditions.

## 2. Experimental

**Figure 1.**
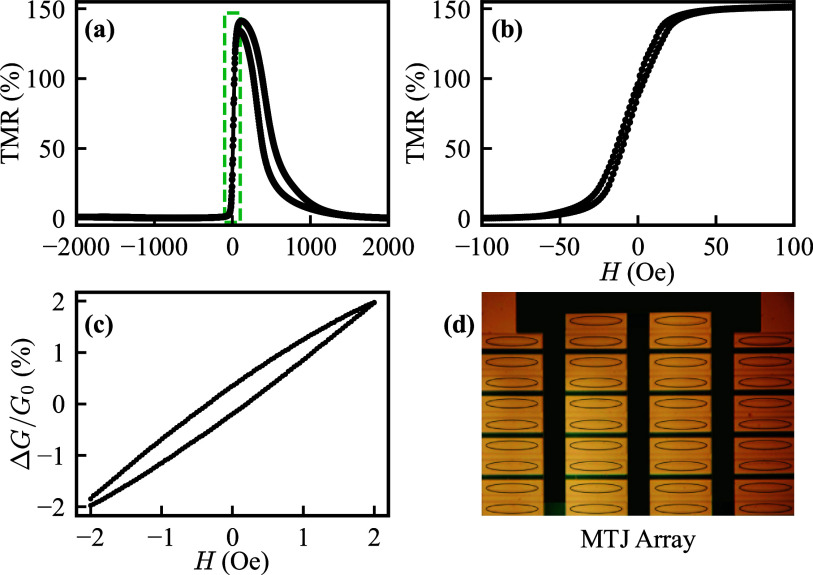
Magnetic characterization of the MTJ sensor. (a) Extended TMR-loop measured up to $\pm$2000 Oe, showing the distinct switching of the free and pinned magnetic layers. (b) Expanded view of the free-layer major loop (dashed box in (a)), highlighting its saturation behavior and coercive field. (c) Representative 2 Oe minor loop. (d) Optical micrograph of the patterned MTJ device.

The MTJ devices were deposited using a high vacuum magnetron sputtering system, with a base pressure of $3\times10^{-8}$ Torr, onto thermally oxidized silicon wafers. The layer structure used was: Ta(5 nm)/Co$ _{50}$Fe$ _{50}$(2)/IrMn(15)/Co$ _{50}$Fe$ _{50}$(2)/Ru(0.8)/Co$ _{40}$Fe$ _{40}$B$ _{20}$(3)/MgO(2)/Co$ _{40}$Fe$ _{40}$B$ _{20}$(30). Following deposition, the stack was patterned using standard photolithography techniques and ion milling to define 160 ${\mu}$m $\times$ 20 ${\mu}\mathrm{m}$ elliptical elements. Electrical contacts were patterned to integrate 38 series-connected individual MTJ devices into a single sensor array (figure 1(d)). This arrangement reduces magnetic noise and increases the sensor output voltage while maintaining safe operating voltages for each individual device, as well as reduces the impact of local defects in the magnetic material for a given device. Post-processing, the devices were annealed for 4 h at 310 $ ^\circ$C under a 4.5 kOe magnetic field along the short axis to define the reference layer direction and promote crystallization of the MgO barrier. In zero field, the free layer’s in-plane magnetization aligns with the long axis due to shape anisotropy. Fields applied along the short axis rotate this magnetization relative to the fixed reference layer, producing a change in MTJ conductance.

Dc magnetotransport measurements were conducted using both a probe station and a Quantum Design™ Physical Property Measurement System^®^ to obtain minor and major conductance-field ($G(H)$) loops for comparison with the ac response. For ac characterization, the sensor array was biased with a 400 ${\mu}$A dc current and positioned within a calibrated field-producing coil inside a Faraday enclosure. The voltage modulation corresponding to the conductance response from an oscillating magnetic field was detected at the excitation frequency using a lock-in amplifier. The ac and dc measurements were performed on separate, nominally identical sensors.

## 3. Results and discussion

### 3.1. Reversible transition

We first characterize the baseline response of the sensor under dc magnetic fields. Figure 1 summarizes the magnetic and transport behavior of the patterned uniaxial MTJ sensor. The extended major-loop in figure 1(a) exhibits two distinct switching events corresponding to the free and pinned layers, confirming that the pinned layer remains stable over the field range relevant to sensor operation. An expanded view of the free-layer reversal, shown in figure 1(b), reveals a tunnel magnetoresistance (TMR) ratio of 153% and a dynamic field range exceeding 15 Oe. A minor loop measured with a 2 Oe maximum field, shown in figure 1(c), demonstrates a clear hysteretic response characteristic of irreversible magnetization processes of the free layer. These irreversible processes correspond to energy-dissipative micromagnetic events, such as domain walls hopping between pinning sites, that require a threshold magnetic energy to overcome local pinning barriers, resulting in the observed hysteresis.

In contrast, reversible processes involve non-dissipative mechanisms, such as elastic domain wall bowing or the coherent rotation of magnetic spins. If the applied field amplitude is sufficiently small, the system lacks the energy required to trigger irreversible jumps. In this low-field regime, the magnetization dynamics are governed strictly by reversible processes, leading to a hysteresis-free response. This transition into a purely reversible phase is highly advantageous for sensing applications, as it minimizes magnetic hysteresis error.

**Figure 2.**
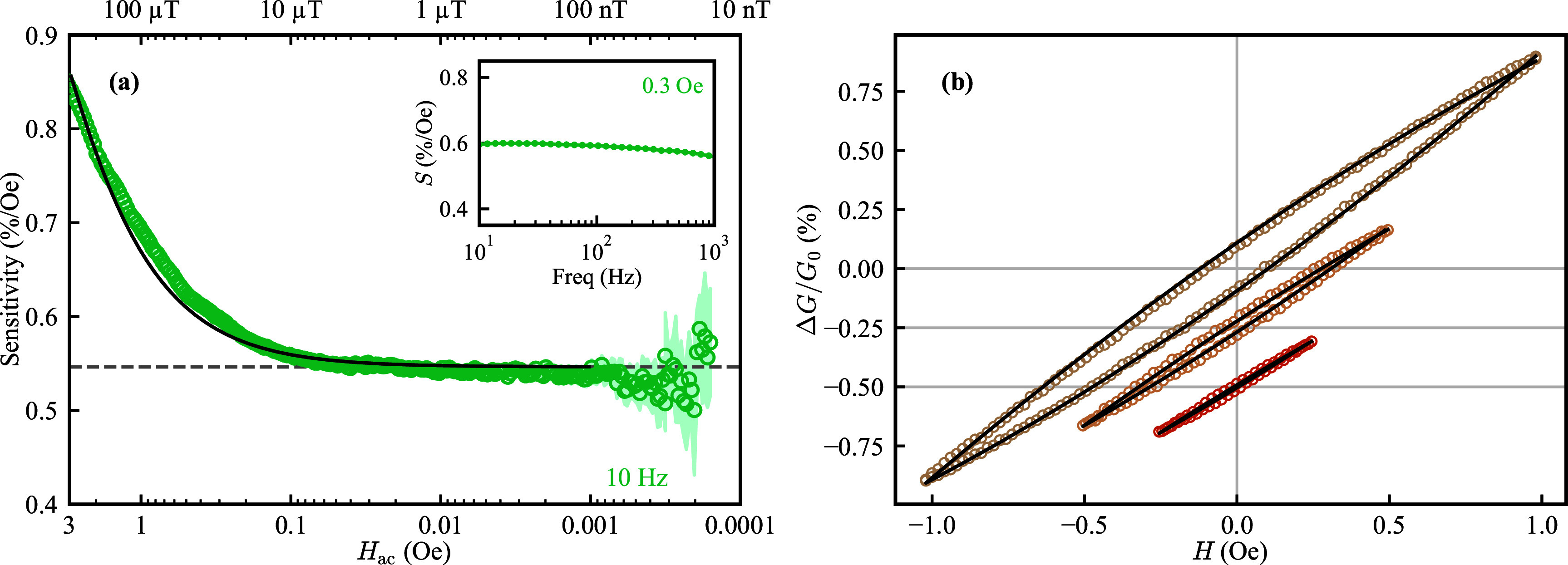
(a) Field- and frequency-dependent sensitivity of the MTJ sensor. The main panel shows the measured sensitivity at a representative frequency (10 Hz) as a function of the ac drive field amplitude ($H_{\mathrm{ac}}$). The sensitivity saturates to a constant value for $H_{\mathrm{ac}}$ < 0.05 Oe. The solid line represents the Jiles–Atherton (JA) fit, with parameters listed in table 1. The inset shows frequency dependence of the sensitivity at $H_{\mathrm{ac}}$ = 0.3 Oe up to 1 kHz. (b) Representative minor *G(H)* loops (offset for clarity) and corresponding JA fits (solid lines) obtained using the same parameter set, showing excellent agreement between experiment and model.

To further illuminate the reversibility transition, we examined the field- and frequency-dependent response of the MTJ sensor, as summarized in figure 2. Figure 2(a) shows the measured sensitivity as a function of the ac drive amplitude $H_\mathrm{ac}$ at a representative frequency of 10 Hz. The sensor’s field sensitivity, in units of %Oe$ ^{-1}$, is defined as

\begin{equation*} S = \frac{1}{G_\mathrm{0}}\frac{\Delta G}{\Delta H},\end{equation*}

where $G_0$ is the zero-field conductance, $\Delta G$ is the conductance change, and $\Delta H$ is the corresponding change in applied field. At the largest drive amplitude of 3 Oe, the sensitivity reaches approximately 0.85%Oe$ ^{-1}$, however as $H_\mathrm{ac}$ decreases to below 0.05 Oe, the influence of irreversible processes diminishes, and the sensitivity saturates to a constant value of about 0.55%Oe$ ^{-1}$. This saturation represents the intrinsic small-signal response governed by the reversible component of magnetization. The inset of figure 2(a) shows that the sensitivity remains nearly frequency independent up to 1 kHz, confirming that the measured response originates from quasistatic magnetization dynamics rather than dynamic losses. At larger fields, nonlinearities in the device response inherently produce higher-order harmonics. These were not included in this part of the study as our focus was strictly on defining the location and magnitude of the linear regime. The influence of these harmonics will be demonstrated later in the manuscript.

Figure 2(b) displays representative minor $G(H)$ loops acquired at several field sweep ranges at or below 1 Oe. As the field amplitude is reduced, the loop area decreases systematically, illustrating the progressive suppression of irreversible magnetization processes. For the smallest sweep field, 0.25 Oe, the loop collapses into a narrow, nearly linear trajectory. The solid lines in both figures 2(a) and (b) are fits obtained using the JA model (to be discussed later) with the same parameter set extracted from the sensitivity data, demonstrating that a single set of parameters consistently reproduces both the amplitude-dependent sensitivity and the shape of the minor hysteresis loops.

### 3.2. JA modeling

The high sensitivity of the MTJ sensor at low magnetic fields enables the acquisition of high-quality data suitable for quantitative model fitting. This allows the JA model to be extended into a low-field regime that is not typically accessible. Furthermore, the model can be formulated directly in terms of the MTJ conductance, which is directly proportional to the free-layer magnetization. In the standard formulation, the total magnetization $M$ is expressed as the sum of an irreversible component $M_\mathrm{irr}$ and a reversible contribution proportional to the difference between $M_\mathrm{irr}$ and the anhysteretic magnetization $M_\mathrm{an}$:

\begin{equation*} M = M_\mathrm{irr} + c\left(M_\mathrm{an} - M_\mathrm{irr}\right),\end{equation*}

where the dimensionless parameter $c$ ($0 \unicode{x2A7D} c \unicode{x2A7D} 1$) is a mixing parameter that adjusts the strength of each contribution. Physically, these magnetization contributions represent dissipative energy events where irreversible domain wall displacement across pinning sites occurs, and conserved energy motion dominated by coherent spin rotation and elastic domain wall bowing, respectively. $c$ should not be interpreted as a literal fraction of spins or domains participating in reversible motion, but rather as a phenomenological parameter governing the balance between the reversible and irreversible responses. The irreversible component evolves according to

\begin{equation*} \frac{\mathrm{d}M_\mathrm{irr}}{\mathrm{d}H} = \frac{M_\mathrm{an} - M} {k \delta - \alpha \left(M_\mathrm{an} - M\right)},\end{equation*}

where $k$ characterizes the average pinning strength of domain walls and is related to coercivity, $\alpha$ is a mean-field coupling constant that accounts for interdomain interactions, and $\delta = \pm 1$ distinguishes increasing and decreasing field sweeps. The anhysteretic magnetization $M_\mathrm{an}$ is given by a Langevin function of the effective field:

\begin{equation*} M_\mathrm{an} = M_\mathrm{s}\! \left( \coth\!\left(\frac{H + \alpha M}{a}\right) - \frac{a}{H + \alpha M} \right),\end{equation*}

where $M_\mathrm{s}$ is the saturation magnetization and $a$ defines the magnetic softness of the material, changing the curvature of the anhysteretic curve. Collectively, these parameters govern the shape and reversible slope of the hysteresis loop.

Because the tunneling conductance in an MTJ depends on the relative magnetization orientation of the free and pinned layers, the normalized conductance change $g(H) = \Delta G(H)/G_0$, where $\Delta G(H) = G(H) - G_0$ represents the change in conductance from its zero-field value $G_0$, can be taken as proportional to the normalized magnetization $M(H)/M_\mathrm{s}$. This correspondence allows the JA model to be expressed directly in terms of experimentally accessible conductance quantities. Defining the normalized anhysteretic conductance as $g_\mathrm{an}(H) = \Delta G_\mathrm{an}(H)/G_0$, and the irreversible component $g_\mathrm{irr}(H) = \Delta G_\mathrm{irr}(H)/G_0$, the normalized conductance-field relationship becomes

\begin{equation*} g = g_\mathrm{irr} + c \left(g_\mathrm{an} - g_\mathrm{irr}\right),\end{equation*}

which mirrors equation (2). Substituting this conductive form into equation (3) yields a model that can be fit directly to the measured $G(H)$ loops and sensitivity data without requiring separate magnetization measurements. The differential sensitivity in the JA framework is

\begin{equation*} S = \frac{\mathrm{d}g}{\mathrm{d}H} = c\frac{\mathrm{d}g_\mathrm{an}}{\mathrm{d}H} + \left(1-c\right) \frac{g_\mathrm{an} - g} {\delta k - \tilde{\alpha}\left(g_\mathrm{an} - g\right)}\end{equation*}

where $\mathrm{d}g_\mathrm{an}/\mathrm{d}H$ represents the reversible slope of the anhysteretic curve and the second term captures irreversible pinning effects. We also introduce a scaled coupling coefficient using the conductive saturation $g_\mathrm{s}$, the total percent change in conductance from zero field:

\begin{equation*} \tilde{\alpha} = \alpha\frac{M_s}{g_s}.\end{equation*}

This properly accounts for the change in units and should be kept in mind when comparing $\alpha$ values across different sensors. Equation (6) provides a direct link between the measured sensitivity and the underlying magnetic parameters $a$, $k$, $c$, $\alpha$, and $g_\mathrm{s}$, enabling quantitative interpretation of the transition from hysteretic to reversible behavior observed experimentally in figure 2.

The JA model contains several coupled parameters that must be determined self-consistently from the experimental data. To minimize parameter degeneracy and improve the robustness of the fit, we constrained the conductive saturation $g_\mathrm{s}$ and the small-signal reversible sensitivity $S_\mathrm{rev}$ to the experimentally measured values, leaving the remaining parameters ($a$, $k$, $\tilde{\alpha}$, and $c$) to be optimized globally. The fitting procedure was performed simultaneously on the sensitivity data and the minor $G(H)$ loops shown in figure 2(b), ensuring that a single parameter set consistently reproduced both the amplitude-dependent response and the detailed hysteresis behavior. The sensitivity was fit by stepping each increase in $H_{\mathrm{ac}}$ and evaluating the average slope at each point, which holds for low fields far from saturation. To account for experimental variations such as thermal drift, device-to-device deviation or calibration offsets, as well as the approximation in the sensitivity fit, a scaling factor was allowed for the dc measurement sets.

The fitted parameters listed in table 1 should be interpreted as effective fit parameters rather than intrinsic material constants. Although the JA model was originally developed for bulk ferromagnets and magnetic alloys [25, 26], here it is used purely as a phenomenological framework for thin-film MTJ devices. The standard JA formulation does not include the dominant anisotropy and demagnetization energies that govern the macrospin response of our thin-film CoFeB free layer. Consequently, the extracted parameters do not reproduce the full major loop and are not intended to do so. Instead, the JA model functions here to capture the progressive collapse of hysteresis and the emergence of reversible rotation strictly under small applied fields. Although extended JA models with explicit anisotropy terms exist, implementing them would obscure the primary point of our analysis, which is to demonstrate that detectability is systematically overestimated when sensitivity is inferred from large-field behavior rather than from the low-field regime. Within this scope, the fitted parameters reliably track the evolution of loop area and sensitivity, providing a consistent and physically transparent description of the measured $G(H)$ response.

**Table 1.** Fitted Jiles–Atherton parameters with reported uncertainties derived from the covariance matrix.

Parameter	Value
$g_\mathrm{s}$ (%)	59
$a$ (Oe)	3.87 $\pm$ 0.02
$k$ (Oe)	33.6 $\pm$ 0.14
$\tilde{\alpha}$ (Oe)	0.115 $\pm$ 0.004
$c$ (dimensionless)	0.1064 $\pm$ 0.0004

$S_\mathrm{rev}$ (%Oe$ ^{-1}$)	0.546

### 3.3. Hysteresis loss

**Figure 3.**
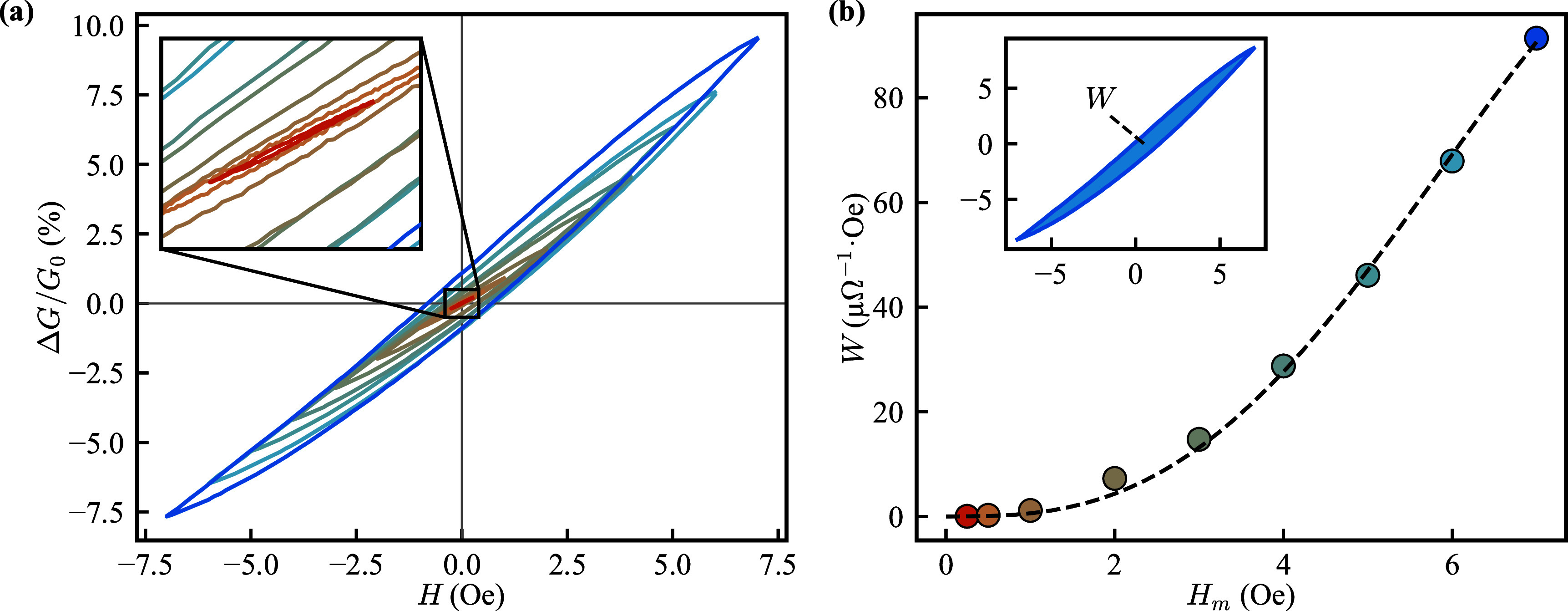
Transition to non-hysteretic behavior at low magnetic fields. (a) Minor conductance-field loops measured for several dc field amplitudes, from 7.5 Oe down to 0.25 Oe, showing the progressive collapse of the hysteretic opening as the field decreases. (b) The hysteresis loss *$W$*, obtained from the integrated loop area, plotted as a function of the maximum field amplitude $H_{m}$. The loss decreases rapidly with decreasing field and approaches zero in the reversible regime. The dashed line represents a fit based on Rayleigh-type scaling, $W = DH_{m}^3 + EH_{m}^4$.

To further clarify the small-field regime relevant to detectability, we examine minor-loop behavior that offers a complementary view of energy dissipation in the free layer, independent of the JA modeling range. Figure 3(a) shows a sequence of minor $G(H)$ loops measured as the maximum field $H_\mathrm{m}$ was systematically reduced from 7.5 Oe to 0.25 Oe. The progressive collapse of the loop area with decreasing amplitude reflects the diminishing contribution of irreversible domain motion predicted by the JA model.

To quantify this behavior, the hysteresis loss $W$, obtained by integrating the loop area, is plotted in figure 3(b) as a function of $H_\mathrm{m}$. The loss decreases rapidly as $H_\mathrm{m}$ is lowered, eventually approaching zero near the field amplitude where the sensitivity in figure 2(a) reaches its plateau value. The dotted line represents the fit, which follows a Rayleigh-type scaling,

\begin{equation*} W\left(H_\mathrm{m}\right) = DH_\mathrm{m}^3 + EH_\mathrm{m}^4,\end{equation*}

characteristic of hysteretic systems [27]. This trend confirms that the energy dissipated per cycle, proportional to irreversible domain-wall motion, collapses as the free layer transitions into the fully reversible regime.

### 3.4. Impact on detectability

Having established the intrinsic reversible response through the JA framework, we next assess its practical impact on sensor performance by evaluating the magnetic noise floor. Although the current MTJ array is not an ideal sensor due to its finite hysteresis, the extracted reversible sensitivity remains high. Detectability is defined as

\begin{equation*} S_H^{1/2}\left(f\right) = \frac{1}{S} \frac{S_V^{1/2}\left(f\right)}{V_\mathrm{bias}},\end{equation*}

where $S_V(f)$ is the power spectral density (PSD), $V_\mathrm{bias}$ is the bias voltage across the MTJ during the noise measurement and $S$ is defined in equation (1). As an independent validation of our framework and to provide deeper insight into the low-field dynamics, we performed these detectability measurements under a small ac magnetic field excitation at a fixed frequency. This excitation field was calibrated using a commercial fluxgate magnetometer for accuracy and generated a distinct reference peak in the PSD response. By resolving the magnitude of the sensor response at the same frequency, we can directly extract the sensor sensitivity and map the voltage noise floor to an absolute magnetic field equivalent. This ac-excitation technique is well-established in the literature [28, 29] as a high-fidelity method for direct detectability characterization.

**Figure 4.**
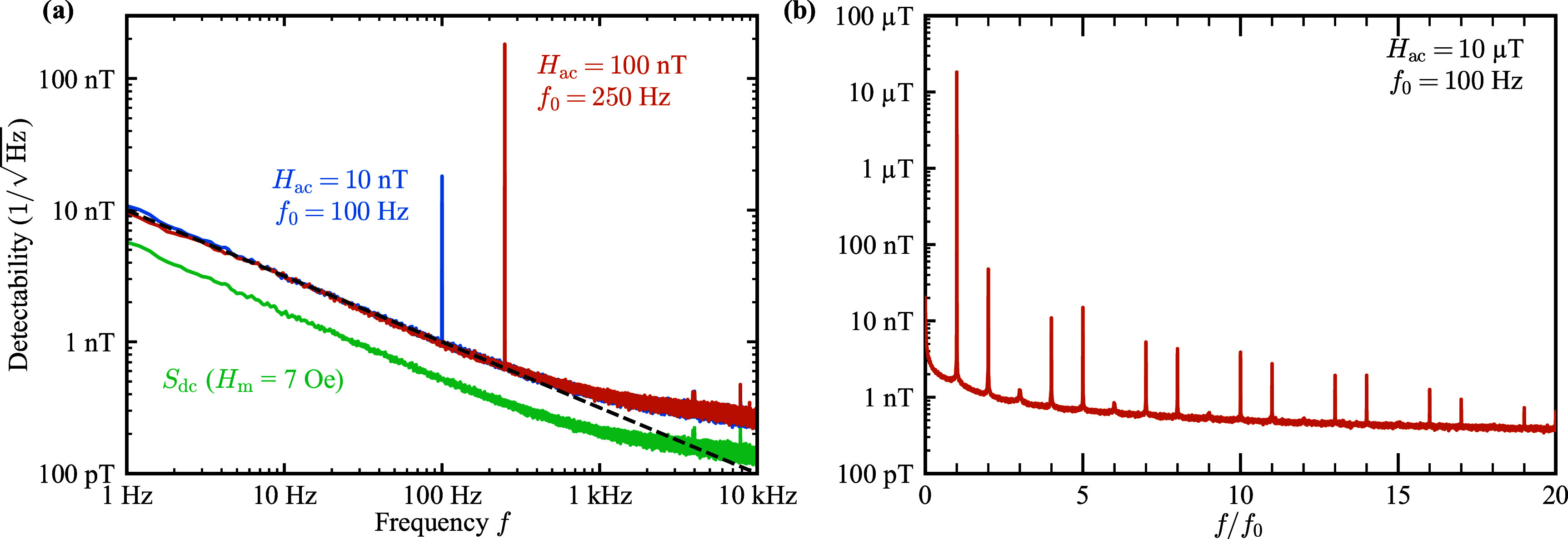
Characterization of linear and nonlinear magnetic response regimes via ac-excited detectability measurements. (a) Low-field detectability spectra under ac excitation amplitudes and frequencies $H_\mathrm{ac} = 10\,\mathrm{nT}$ at $f_0 = 100\,\mathrm{Hz}$ (blue) and $H_\mathrm{ac} = 100\,\mathrm{nT}$ at $f_0 = 250\,\mathrm{Hz}$ (orange). The baseline noise floors of both low-field ac-excited spectra collapse identically onto the true intrinsic $1/f$ noise floor (dashed black line). The green spectrum is calculated using the larger 7 Oe minor loop sensitivity from the hysteretic regime, which produces an artificially optimistic detectability that cannot be reached at low fields. (b) High-amplitude ac detectability spectrum ($H_\mathrm{ac} = 10\ \mu\mathrm{T}$ at $f_0 = 100\,\mathrm{Hz}$) plotted against normalized frequency $f/f_0$. The sensor response exhibits pronounced harmonics, demonstrating the breakdown of the linear regime at large excitation fields.

Figure 4(a) displays two such noise measurements: one acquired with a 10 nT excitation at 100 Hz, and a second with a 100 nT excitation at 250 Hz. Crucially, both of these excitation amplitudes reside well below the $0.05$ Oe (5 ${\mu}$T) reversibility threshold identified by our JA framework. The resulting field-equivalent spectra are in excellent agreement, showing an identical $1/f$ noise background, a characteristic of MTJs. The intrinsic sensitivity extracted via this direct ac-excitation method is 0.63%Oe$ ^{-1}$, which closely aligns with the convergent low-field 0.55%Oe$ ^{-1}$ value derived from our field-dependent sensitivity measurements. This minor variance may be attributed to differences in the operating conditions, specifically, the noise measurements were conducted at a lower dc bias voltage than the transport loops. Notably, this direct dynamic measurement reinforces the central conclusion of our study, that calculating detectability using conventional, large-field hysteretic metrics (such as the 7 Oe minor loop) artificially inflates the sensor’s performance, underestimating the true noise floor by approximately a factor of two.

Figure 4(b) highlights an alternative perspective that connects the observed reversible-irreversible transition with classic Rayleigh-type scaling. Performing the same ac-excitation detectability measurement at an amplitude slightly above the reversibility threshold yields a different spectral profile, characterized by distinct harmonic peaks at integer multiples of the excitation frequency. These harmonics originate from the non-linear magnetic response of the free layer when driven into its hysteretic regime. By evaluating a Rayleigh polynomial expansion under a purely sinusoidal excitation field $H(t) = H_\mathrm{ac} \sin(2\pi f_0 t)$, the higher-order non-linear terms mathematically map to these higher harmonic frequencies via trigonometric identity expansion. This spectral behavior confirms the non-ideal, distorted response of the sensor when operated within its hysteretic regime, while simultaneously validating that the response converges to a perfectly linear, harmonic-free readout when confined to the intrinsic reversible regime.

## 4. Conclusion

We have demonstrated that uniaxial MTJ sensors undergo a clear transition from hysteretic to fully reversible magnetization dynamics as the excitation amplitude approaches the noise floor. In this regime, the sensitivity converges to a constant value of 0.55%Oe$ ^{-1}$, defining an intrinsic, history-independent response of the free layer. Although the numerical value is device-specific, the convergence to a reduced reversible slope is a general consequence of operating any hysteretic sensor in the small-field limit. A single set of JA parameters consistently reproduces the measured sensitivity and minor-loop evolution, establishing a unified description of the reversible-irreversible crossover.

Because detectability scales inversely with sensitivity, the use of large-field hysteretic slopes systematically overestimates MTJ performance. In the present device, employing even a 7 Oe minor-loop sensitivity overestimates the true magnetic noise floor by approximately a factor of two. The reversible small-field sensitivity therefore defines the physically accessible detectability, aligning with direct low-field measurements and providing a rigorous basis for evaluating ultrasensitive MTJ sensors.

Beyond defining an intrinsic metric for sensor performance, operating in the reversible regime enables a broader form of localized electrical magnetometry. Because MTJ sensors exhibit maximal sensitivity near zero applied field, they are uniquely suited to probe the initial, reversible susceptibility of nearby soft magnetic materials under nanotesla-scale excitation, complementing the regime where conventional bulk magnetometers are least sensitive. In such a configuration, a material positioned in close proximity to the MTJ modifies the local magnetic field through its induced magnetization under small oscillatory excitation. The MTJ converts this excitation into an electrical signal, allowing parameters such as low-field susceptibility, domain-wall pinning strength and mean-field coupling to be extracted through analysis within the JA framework without the need for mechanical modulation. This method transforms the MTJ from a standalone sensor into a high-performance magnetometer capable of efficiently quantifying soft magnetic materials in the nanoscale regime.

## Data Availability

The data that support the findings of this study are openly available at the following URL/DOI: https://doi.org/10.7910/DVN/LSE5NS [30].
